# Artificial light at night weakens body condition but does not negatively affect physiological markers of health in great tits

**DOI:** 10.1242/jeb.249926

**Published:** 2025-07-07

**Authors:** Rachel R. Reid, Neal Dawson, Neil P. Evans, Christopher Mitchell, Jelle Boonekamp, Davide M. Dominoni

**Affiliations:** ^1^ School of Biodiversity, One Health and Veterinary Medicine, Graham Kerr Building, University of Glasgow, Glasgow, G12 8QQ, UK; ^2^ School of Biodiversity, One Health and Veterinary Medicine, Jarrett Building, University of Glasgow, Glasgow, G61 1GH, UK; ^3^ Environment and Sustainability Institute, University of Exeter, Penryn Campus, Penryn, Cornwall, TR10 9FE, UK

**Keywords:** Avian health, Avian physiology, Light pollution, Artificial light, Urbanisation, Avian morphology

## Abstract

Urbanisation brings many novel challenges for wildlife through changes to the natural environment; one of the most unprecedented of these modifications is artificial light at night (ALAN). ALAN has been shown to have profound effects on the behaviour and physiology of many wildlife species, which in turn have negative consequences for fitness and survival. Despite increasing knowledge of the mechanisms by which ALAN can affect health, studies that have investigated this relationship have found contrasting results. This study investigated the impact of ALAN on health biomarkers in 13 day old great tit (*Parus major*) nestlings including malondialdehyde levels (a measure of oxidative damage), antioxidant capacity of plasma, feather corticosterone levels and scaled mass index. Immediately after hatching, broods were either exposed to 1.8 lx of ALAN until day 13 or left unexposed. ALAN treatment significantly reduced scaled mass index but there were no clear negative effects of ALAN on malondialdehyde levels, antioxidant capacity or corticosterone levels. This demonstrates that only certain aspects of health are impacted by early-life ALAN, highlighting the importance of future studies measuring several biomarkers of health when investigating this relationship. Nestlings that fledge the nest in poor body condition have a decreased chance of surviving into adulthood. As urbanisation continues to expand, the negative effects of ALAN on wildlife are likely to become more pronounced. Therefore, it is crucial to gain a better understanding of this relationship.

## Teaser

**Summary:** Exposure to artificial light at night during the developmental period in great tit nestlings causes a decline in body condition but has no clear effect on oxidative stress or feather corticosterone levels.

## INTRODUCTION

Urbanisation is increasing at an unprecedented rate as a result of the continued growth of the global human population. Urbanisation is characterised by extreme modifications of natural habitats which are typically accompanied by novel stressors for native animal and bird species, including the introduction of high levels of chemicals and metals into the ground (Merga et al., 2020; Sharley et al., 2016), increased levels of air (Duh et al., 2008; Gong et al., 2012) and noise pollution (Abbaspour et al., 2015; Yang et al., 2020), high amounts of refuse which include anthropogenic food sources (Murray et al., 2015; Theimer et al., 2015), and increased ambient temperatures (the ‘heat island effect’) (Mathew et al., 2016). One of the most unprecedented of these modifications is light pollution (Bennie et al., 2015; Gallaway et al., 2010) and artificial light at night (ALAN). The global estimates of the spread of ALAN are limited and the impact of ALAN on the environment is not well understood (Gaston et al., 2013). Recent estimates have shown that observable light emissions increased by 49% between 1992 and 2017 which is a faster rate of growth than the human population globally (Gaston and Sánchez De Miguel, 2022). ALAN is derived from various sources including street lighting, advertisements, security lighting, domestic lighting and headlights from vehicles (Gaston et al., 2013). ALAN has been shown to disrupt natural patterns of light through both direct illumination as well as skyglow, where ALAN that is emitted or reflected upwards is scattered by molecules or aerosols in the atmosphere, which spreads ALAN even further and leads to the brightening of the night sky (Gaston et al., 2013; Kouteib et al., 2015).

There are many ways in which ALAN can cause negative consequences for wildlife. The disruption of natural light cycles by ALAN can have profound effects on the behaviour of many wildlife species, impacting reproduction (De Jong et al., 2017; Dominoni et al., 2013), foraging (Dwyer et al., 2013; Santos et al., 2010) and migration (Cabrera-Cruz et al., 2018; Poot et al., 2008). This is because most organisms synchronise their daily and seasonal activities to the light–dark cycles produced by the Earth's rotation (Russart and Nelson, 2018), to optimise the timing of behaviour and physiological responses with prevailing environmental conditions (Dominoni et al., 2013; Russart and Nelson, 2018). For instance, many physiological processes exhibit circadian rhythms, including plasma glucose (Mason et al., 2020), testosterone (Xiao et al., 2021) and glucocorticoid concentrations (Russart and Nelson, 2018; Son et al., 2008), insulin production (Challet, 2015) and immune responses (Labrecque and Cermakian, 2015; Logan and Sarkar, 2012). One mechanism through which ALAN can cause circadian disruption is by changes to activity patterns; for example, diurnal species increasing activity levels during the night. This has the potential to disrupt the sleep–wake cycle, which may have a knock-on effect on physiological processes (Meléndez-Fernández et al., 2023). ALAN has been shown to suppress the nocturnal production of melatonin. Melatonin has an important role in driving biological rhythms and relays important information to the organism about day length (Jones et al., 2015), but melatonin is also a strong antioxidant (Zhang and Zhang, 2014). Despite our knowledge of several mechanisms by which ALAN can have negative consequences for health, the plethora of studies that have investigated this relationship have found contrasting results. The lack of consensus in the field highlights the need for additional high-quality studies to identify trends, mechanisms or context-specific effects. This growing body of evidence could eventually support a comprehensive meta-analysis, which to our knowledge has not yet been conducted.

There are many studies that have shown a negative relationship between ALAN and health, particularly in human subjects. Studies that have focused on shift workers have documented that exposure to high-intensity ALAN increased the risk of cancer, immune suppression, heart disease and metabolic dysregulation (Jones et al., 2015; Russart and Nelson, 2018). Negative impacts of ALAN exposure have also been shown in wildlife; for example, ALAN decreased immune function in Siberian hamsters (*Phodopus sungorus*) (Bedrosian et al., 2011) and great tit (*Parus major*) nestlings (Ziegler et al., 2021). In the latter study, it was also documented that exposure of the nestlings to ALAN resulted in 49% lower levels of melatonin during the night than the control birds and it is likely that this would have had effects on other physiological processes (Ziegler et al., 2021).

There are also studies that have shown little or no effect of ALAN on health. For example, ALAN was found to have no effect on the level of oxidative stress markers in freshwater crustacean shredders (Czarnecka et al., 2022). However, rats exposed to constant light at night exhibit increased oxidative stress levels and impaired antioxidant enzyme activity (Cruz et al., 2003). Another study reported that ALAN had no effect on telomere shortening of great tit nestlings and only a weak relationship with body condition (Grunst et al., 2020) but this result is in contrast with other studies that show a strong influence on the body condition of nestlings (Ferraro et al., 2020; Raap et al., 2016c). Together, these findings show that the relationship between ALAN and health may be complex and could be dependent on a variety of factors including species, life stage and the health biomarker measured.

Despite the increasing interest in the relationship between ALAN and wildlife health, there are limited field studies that have exposed birds to manipulated levels of ALAN to investigate the impact on health biomarkers (Dominoni et al., 2021; Grunst et al., 2019, 2020; Raap et al., 2016a,b,c; Ziegler et al., 2021). This type of experimental study is of particular interest in regard to exposure to ALAN during early development as this is a sensitive stage in a bird's life. Exposure to stressful conditions during early development has been shown to affect growth, metabolism, immunocompetence and sexual attractiveness later in life (Lindström, 1999). Most of the studies in the field have focused on adult birds (De Jong et al., 2015; Dominoni et al., 2013; Kernbach et al., 2020; Ouyang et al., 2017; Raap et al., 2017b). The few studies examining the developmental period typically exposed nestlings to ALAN for only 2–7 nights (Grunst et al., 2020; Raap et al., 2016b, 2018). In contrast, we exposed nestlings to ALAN for the first 13 days of their life, covering the bulk of their development. To our knowledge, the only other study with a similar exposure period is that of Dominoni et al. (2021). This study was able to demonstrate that impacts of ALAN on feather corticosterone were population specific, with urban birds experiencing increased levels of feather corticosterone and forest birds experiencing reduced levels of feather corticosterone when exposed to ALAN (Dominoni et al., 2021).

The present study tested the hypothesis that exposure of great tit nestlings to dim ALAN throughout the night for the first 2 weeks of their life, until close to fledging, would have negative impacts on the nestlings' health. Health is a multivariate trait; therefore, it can be complex to measure and understand. We aimed to address this complexity by measuring multiple markers of health to provide a more comprehensive assessment of how it is being impacted by ALAN. Most field studies investigating the physiological and health impacts of ALAN on birds have measured only one or two biomarkers (Dominoni et al., 2013, 2021; Kernbach et al., 2020; Raap et al., 2016c, 2017a, 2018), with a few exceptions (Ouyang et al., 2017; Raap et al., 2016b; Grunst et al., 2020). Here, we measured four biomarkers of health including scaled mass index (SMI), antioxidant capacity of plasma (OXY), and malondialdehyde (MDA) and feather corticosterone (fCORT) levels. SMI is a proxy of body condition, providing a measure of the fat content or nutrient reserves of an animal (Peig and Green, 2009; Schamber et al., 2009), and can be influenced by both exposure to stressful conditions and nutritional deprivation (Labocha and Hayes, 2012). Oxidative stress arises when pro-oxidants overwhelm antioxidants, which leads to the highly reactive oxygen species which can damage the organism's own tissues. Oxidative stress has been linked to many important processes including senescence, expression of sexual ornamentation and sperm performance as well as survival and reproduction (Sepp et al., 2012; Speakman et al., 2015). It has been highlighted in various reviews that to understand oxidative stress, it is beneficial to have at least one measure of oxidative damage as well as a measure of antioxidant defence (Costantini, 2019; Costantini and Verhulst, 2009; Speakman et al., 2015). The measure of oxidative damage used in the current study was MDA, which is a product of lipid peroxidation. OXY was also used as a measure of antioxidant defence as it integrates the total non-enzymatic antioxidant defence that is found in the organism's plasma. Lastly, CORT is the main glucocorticoid and the effector of the hypothalamic–pituitary–adrenal (HPA) axis in birds. CORT plays an important role in the regulation of physiological and behavioural responses to stressors (Bonier, 2012). Most studies that have focused on urban endocrine ecology have used measures of baseline and stress-induced plasma CORT concentrations. Plasma CORT, however, only provides information about the short-term endocrine state of the individual. Feather CORT concentrations give a longer-term integrated measure of endocrine state, as CORT is deposited from the bloodstream into the feather structure during feather growth. Therefore, this measure integrates both baseline and stress-induced CORT concentrations over time (Romero and Fairhurst, 2016), providing the HPA status of a nestling from birth until the time of sampling. The biomarkers measured in this study were selected as research has shown that they are regulated by circadian rhythms and are susceptible to circadian disruption (Borniger et al., 2014; Budkowska et al., 2022; Eikenaar et al., 2020; Froy, 2010; Hardeland et al., 2003; Hillebrecht et al., 2024; Sani et al., 2006). This increases the likelihood that these biomarkers may be particularly sensitive to ALAN exposure.

To address our overall hypothesis this study sought to address the following questions. (1) Does ALAN have an impact on the body condition of great tit nestlings? We predicted that there would be a negative effect of ALAN on body condition, as suggested in previous studies (Ferraro et al., 2020; Raap et al., 2016c). (2) Does ALAN impact fCORT levels of great tit nestlings? We predicted that ALAN would increase fCORT levels. (3) Does ALAN impact oxidative stress status of great tit nestlings through effects on MDA and OXY levels? We expected that ALAN would result in increased oxidative stress, and this would be evidenced either by increased oxidative damage, i.e. MDA, or by reduced antioxidant defence, i.e. OXY, or a combination of the two factors.

## MATERIALS AND METHODS

### Study sites

**Fig. 1.**
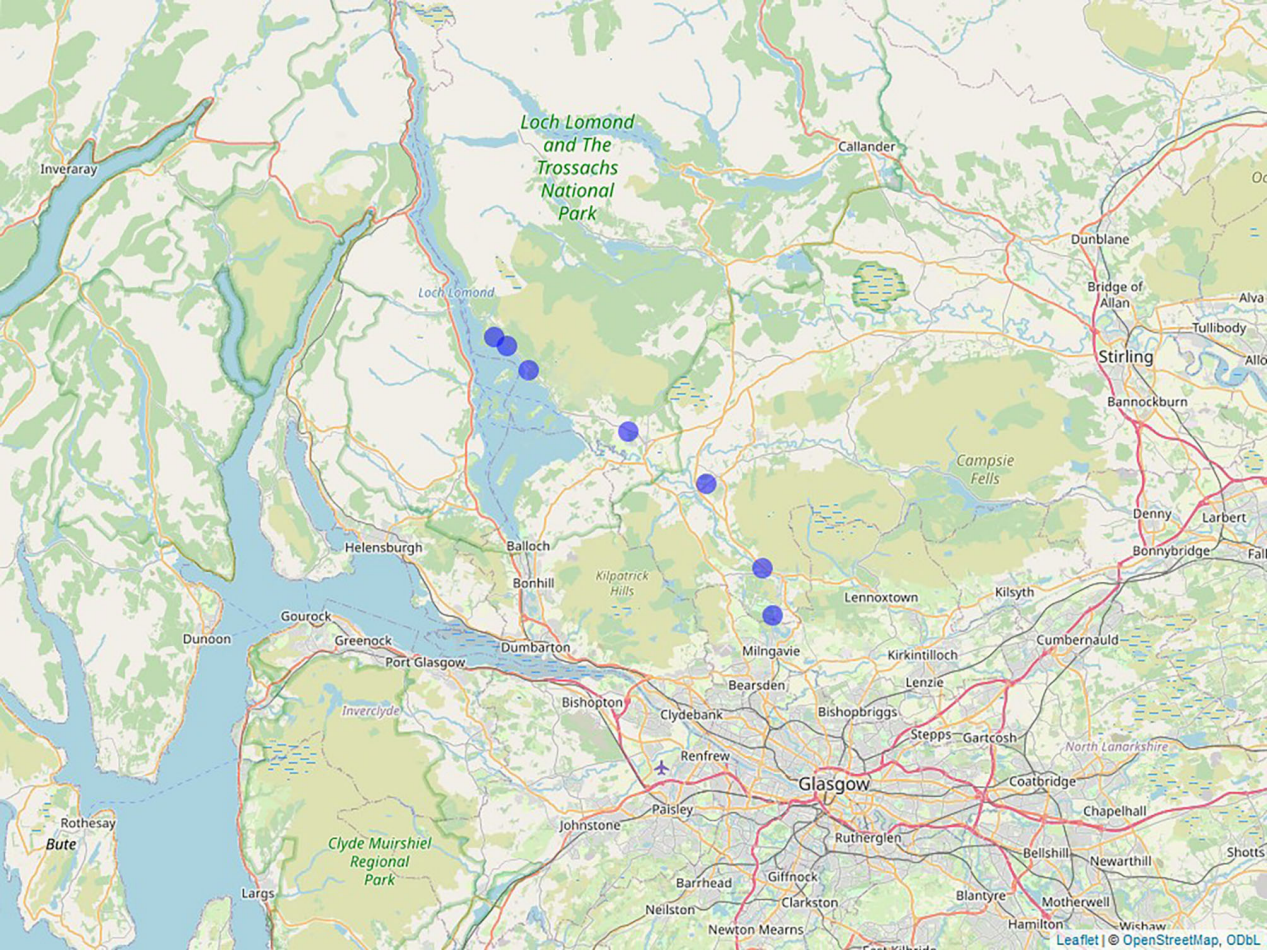
**Map showing study sites used for the field experiment, spanning the rural–urban gradient.** The map was created in R studio using the package ‘Leaflet’ and the function ‘Mapview’ (https://CRAN.R-project.org/package=mapview). Map data obtained from OpenStreetMap contributers, licensed under ODbL. Study sites are indicated by blue dots.

The research took place in seven forest sites, with low anthropogenic disturbance, in the West of Scotland (Fig. 1), between April and June 2021. The exact location of the study sites can be found in Table S1. At each site, existing nest boxes (Woodcrete Schwegler boxes, 17×26×18 cm, hole size 32 mm) were used. Nest boxes used by the study species, *Parus major* Linnaeus 1758, were monitored weekly during the reproductive period and all reproductive activities recorded. Near the expected hatching date, the nest boxes were checked every other day to identify exact hatching dates.

### Ethics statement

All work involving feather clipping was conducted under a British Trust for Ornithology licence (12138) issued to D.M.D. Permission for bird ringing was granted by the British Trust for Ornithology with licences to D.M.D (permit number: 6822). All blood samples were collected under the project licence CPP20193834 issued to D.M.D.

### Manipulation of ALAN

Upon hatching, whole broods were either exposed to ALAN (experimental group) or left unexposed (control group). Nest boxes were allocated to either an experimental or control group alternately, based on their brood size and hatching date. For each nest box (control or experimental), the entire brood, including the original nest cup, was transferred into a new nest box equipped with a single broad-spectrum, cool-white LED bulb attached to the nestbox's ceiling. This bulb was powered by a 12 V, 3.2 Ah battery that sat outside the nest box on the forest floor. A photometer (LI-200A, Li-COR Environmental, UK Ltd, Cambridge, UK) was used to calibrate all lights to a standardised intensity of 1.8 lx (±1 lx), which has been shown to simulate the light in an urban environment (Dominoni et al., 2013). A sensor (Bioelectronics Department, University of Glasgow) was attached to the outside of the battery box and turned the light on or off depending on the ambient light intensity, to ensure that the experimental light manipulation only occurred at night-time. The control nest boxes were also fitted with an LED light on the inside of the nest box roof, but these lights remained turned off. Fourteen experimental nestboxes and 15 control nestboxes were used. The exact allocation of control and experimental boxes to the study site can be found in Table S1.

### Sample collection

To measure physiological biomarkers, blood and feather samples were collected 13 days after the day the first chick in a brood hatched. For every nest, four nestlings were removed from the brood for sampling. All nestlings were first placed in a bird bag, and four were then selected. This was consistently applied across both control and ALAN nest boxes to reduce the risk of bias. To obtain blood samples, the brachial vein was punctured with a sterile needle, and blood was collected into a 75 μl capillary tube. Our primary aim was to ensure that no more than 1% of each chick's body mass was taken, while still attempting to fill the tube whenever possible. Blood was immediately dispensed into a prelabelled 1.5 ml Eppendorf tube. The samples were kept on ice when out in the field. Within 4 h of collection, the samples were spun for 10 min at 10,000 rpm to separate the plasma from the red blood cells. The plasma was then pipetted into a new 0.5 ml Eppendorf tube. Both the red blood cells and plasma were then stored in a −80°C freezer until further analysis.

Two tail feathers no more than 20 mm in length were clipped from each of the four nestlings and transferred into a 1.5 ml Eppendorf tube prelabelled with a unique ID. The feathers were stored in a dry place at room temperature until further analysis.

Every nestling in each nest box was also ringed by a trained ringer. The tarsus length, body mass and wing length of each nestling were also recorded.

In total, we sampled 107 nestlings. For oxidative stress markers (OXY and MDA), we collected samples from 54 nestlings in control nest boxes and 53 nestlings in ALAN nest boxes. For fCORT analysis, we sampled 31 nestlings from control boxes and 30 from ALAN boxes. Differences in sample size across analyses were due to limited sample availability for certain metrics.

### Scaled mass index

Body condition was calculated for each individual using SMI, which standardises body mass to a specific fixed linear measurement of the organism, in this case tarsus length, as per Eqn 1 (taken from Peig and Green, 2009):
(1)

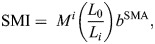
where *M_i_* is body mass and *L_i_* is tarsus length for individual, *i*. The scaling exponent *b*^SMA^ was estimated using a standardised major axis (SMA) regression of log-transformed *M_i_* on log-transformed *L_i_.* This was calculated using the ‘sma’ function from the ‘smatr’ package in R (Warton et al., 2012).

*L*_0_ represents the mean tarsus length of the study population, and SMI is the predicted body mass for an individual when their tarsus length is standardised to *L*_0_ (Peig and Green, 2009).

### DNA extraction

DNA was extracted from the red blood cells using a Puregene kit (Qiagen, Hilden, Germany), following the manufacturer’s protocol. DNA concentration and quality were quantified using a Nanodrop-8000 spectrophotometer (Thermo Fisher Scientific, Waltham, MA, USA). DNA integrity was checked in a subset of 29 samples, using an Agilent 2200 TapeStation (Agilent, Santa Clara, CA, USA). Genomic DNA samples were stored at −80°C.

### Molecular sexing

Molecular sexing of the nestlings was carried out using RT-PCR with primers P2 and P8, as described previously (Griffiths et al., 1998).

### Measuring MDA levels in plasma

Plasma concentrations of MDA were determined using high-performance liquid chromatography (HPLC) as described previously (Nussey et al., 2009). In summary, an aliquot of plasma was transferred to a reaction tube pre-loaded with 20 µl of butylated hydroxytoluene (BHT); 40 µl of thiobarbituric acid (TBA) and 160 µl of phosphoric acid were added and the reaction tubes vortexed for 5 s. The sample was then placed in a dry heat bath (100°C) for 1 h, before being cooled on ice. Once the reaction tube had cooled, 160 µl of butanol was added and the tube vortexed for 10 s. The sample was then centrifuged at 4°C at 12,000 ***g*** for 3 min. An aliquot (60 µl) of the upper phase which contained MDA-TBA adducts was drawn off and placed in a HPLC vial; 20 µl of the prepared sample was injected into a HPLC system (Agilent 1200 series) fitted with a Hewlett-Packard Hypersil 5 µl ODS 100×4.6 mm column and a 5 µl ODC guard column (Thermo Fisher Scientific) kept at 37°C. The mobile phase was methanol/buffer (40:60 v/v) at a flow rate of 1 ml min^−1^. The buffer was 50 mmol l^−1^ potassium monobasic phosphate, adjusted to pH 6.8 using 5 mol l^−1^ KOH. Fluorescence detection was then performed using an Agilent 1260 Infinity III LC HPLC system at 515 nm excitation and 553 emission. The MDA concentrations were calibrated using an external standard of 1,1,3,3-tetraethoxypropane (TEP) serially diluted with 40% ethanol. The intra-plate coefficient of variation (CV) for this assay was calculated using 12 standards run in duplicate for the calibration curve, yielding an intra-plate CV of 4.16%. Repeatability between plates could not be directly assessed because of limited sample availability. However, this assay has been demonstrated to be repeatable in previous analyses conducted in the same laboratory (Bodey et al., 2020; Nussey et al., 2009).

### Measuring OXY levels

OXY was measured in triplicate using an OXY-Adsorbent test (Diacron International, Grosseto, Italy) following the manufacturer's instructions with modifications. A 2 µl sample of plasma was first was diluted 1:100 and then 5 µl of the diluted sample was added to the plate along with 190 µl hypochlorous acid (HOCl; an endogenously produced oxidant). Once the oxidant solution was added, the plate was incubated at 37°C for 10 min. After incubation, 5 μl of chromogen was added to each of the wells. The plate was read using a SpectraMax Plus spectrophotometer (Molecular Devices, San Jose, CA, USA) at a temperature of 37°C and a wavelength of 505 nm. The results of the OXY-Adsorbent test were expressed as μmol of HOCl ml^−1^ of sample. Repeatability was calculated as *R*^2^=0.67 (*N*=15). Repeatability was calculated by repeating every step of the protocol for 15 samples; a linear mixed effects model (LMM) was run using sample ID as a random effect to account for repeated measures within individuals. Repeatability was then calculated as the ratio of the random intercept variance to the total variance. The inter-plate CV calculated using these same 15 samples was 10.62% and the intra-plate CV was 7.03%, which falls in the range reported by Costantini (2011).

### Measuring fCORT levels

Feather samples were washed (10 min) in an orbital shaker once with 1 ml of 20% methanol and twice with UltraPure water, before being left to air dry. This washing will remove any contamination and dirt as well as CORT from other sources such as faeces or preening oils from the surface of the feathers. Once dried, the feathers were cut into lengths of less than 5 mm and weighed. Any samples that weighed <1 mg were excluded. All remaining samples weighed between 1 and 26 mg. To extract CORT from the feathers, 2 ml of HPLC-grade methanol was added to each feather followed by incubation at 52°C in an orbital shaker at 175 rpm for 19 h. After incubation, 1 ml of methanol was removed into a 12×17 mm borosilicate glass tube and dried in a sample concentrator (Thermo Savant SC210A Speedvac concentrator). The samples were reconstituted in 150 μl of assay buffer in a multi-vortexer (SMI, Newmarket, UK) for 10 min. The CORT concentration for each sample was determined using a commercial ELISA kit (Cayman Chemical Corticosterone ELISA Kit, item no. 501320, Ann Arbor, MI, USA) following the manufacturer's instructions.

All samples were run in duplicate, and samples from the same nest boxes were split across different plates to avoid nest effects. Eight standards were run in duplicate on each plate to generate a standard curve and assess plate sensitivity. Optical density was measured at a wavelength between 405 and 420 nm using a LT-4500 absorbance plate reader (Labtech International, Heathfield, East Sussex, UK). Assay Zap computer software (Biosoft, Acropolis Computers Ltd, Cambridge, UK) was then used to calculate the feather CORT concentration. The results were corrected against the mass of the feathers, following previous studies (Dominoni et al., 2021). Intra-plate CV was calculated using the eight standards which were run in duplicate on each plate, and averaged 7.085%. There was limited sample size to calculate repeatability between plates, but this assay has previously been validated in the same laboratory (Dominoni et al., 2021).

### Statistical analysis

All analysis were performed in R (v.4.0.4; http://www.R-project.org/).

#### Effects of ALAN on SMI, OXY, MDA and fCORT

The effects of ALAN on the various health biomarkers measured were examined using LMMs run with the R package ‘lme4’ (Bates et al., 2015). The models were run separately for SMI, MDA, OXY and fCORT. Sex, treatment, brood size and date of sampling were included in the model as predictors. Date of sampling, which refers to the date on which biological samples were collected, was converted into a Julian date so that it could be added to the model as a continuous variable. We also included Julian date as a quadratic term. Treatment×Sex was included as an interaction effect because males and females differ in size, with males potentially outcompeting females for food (Michler et al., 2010; Teather, 1992), which may result in sex-specific treatment responses. Treatment×Date of sampling was also included as an interaction because phenological changes throughout the breeding season can impact reproductive success as a result of phenological mismatch with insect prey (Grimm et al., 2015; Grüebler and Naef-Daenzer, 2010). Birds may therefore respond differently to the treatment depending on the time of breeding.

We included nest box ID nested into study site as a random effect in all models, to take into account the non-independency of samples collected from the same brood, and from broods at the same site. In all models excluding the one where SMI was used as the response variable, SMI was included as a continuous predictor and Treatment×SMI was included as an interaction effect. This interaction was included because birds in poorer body condition may be more sensitive to physiological changes induced by the treatment. Backward selection was always completed for every model using likelihood ratio tests (LRTs) with the ‘drop1’ function in the ‘lme4’ package. To do this, we began with a full model that included all of the relevant main effects and interactions. We sequentially removed non-significant interaction terms, starting with the interaction that had the highest *P*-value. All main effects were retained in the final model, even if non-significant. This approach is common in statistical modelling and the removal of non-essential interactions which may absorb variance helps to prevent overfitting and reduce model complexity. This method also allows for more accurate model estimates for all variables of interest (Johnson and Omland, 2004; Lewis et al., 2011).

*Post hoc* analysis using a Tukey test was run for the MDA LMM model to investigate the pairwise interactions between sex and treatment using the ‘emmeans’ package (https://CRAN.R-project.org/package=emmeans).

#### The effect of OXY on MDA

To examine whether there was an effect of OXY on MDA levels, a LMM was run for MDA where OXY was included as a continuous predictor. A three-way interaction between Treatment, Sex and OXY was also tested in this model.

#### The effects of physiological biomarkers on SMI

To examine the effects the physiological biomarkers measured in the study had on SMI, a LMM was run and included OXY and MDA as continuous predictors. We ran a separate model where fCORT was included as a continuous predictor as this was only known for 61 out of 107 samples. Interaction effects of OXY, MDA and fCORT with treatment were also included in these models as we were interested in whether treatment causes physiological changes that could in turn affect body condition. We also performed a Pearson's correlation using the ‘cor’ (Guo et al., 2024) package to assess relationships between the four biomarkers.

## RESULTS

### Does ALAN impact body condition?

**Fig. 2.**
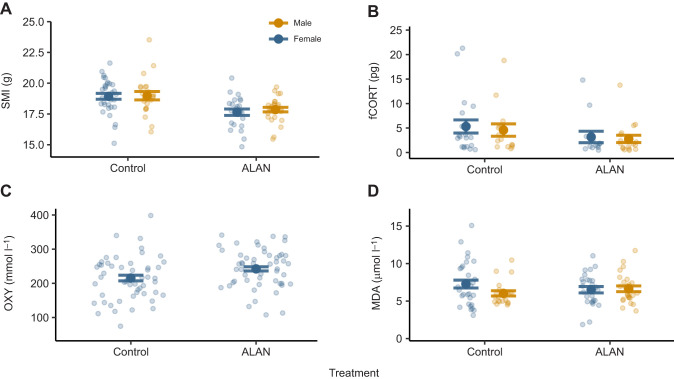
**Impact of treatment group on health biomarkers in male and female great tit nestlings.** Each panel shows means±s.e.m. for control and artificial light at night (ALAN) treatment groups, with raw data points plotted semi-transparently and coloured by sex. (A) Scaled mass index (SMI); sample sizes: ALAN *n*=24 female, *n*=28 male; control *n*=33 female, *n*=22 male. (B) Feather corticosterone (fCORT); sample sizes: ALAN *n*=13 female, *n*=18 male; control *n*=20 female, *n*=15 male. (C) Antioxidant capacity of plasma (OXY), adjusted for experimental plate effects using residuals from the linear mixed model (LMM); sample sizes: ALAN *n*=27 female, *n*=27 male; control *n*=33 female, *n*=25 male. As the model indicated a significant effect of treatment only (no significant sex effect or interaction), data are not separated by sex in this panel to better highlight the treatment effect. The data shown therefore represent both sexes combined. (D) Malondialdehyde (MDA); sample sizes: ALAN *n*=27 female, *n*=27 male; control *n*=33 control, *n*=25 male.

Great tit nestlings exposed to ALAN, regardless of sex, had a significantly lower SMI than controls (Table 1, Fig. 2A).

**Table 1.** The output of the lmer model with the values from the likelihood ratio test (LRT), where scaled mass index (SMI) was used as the response variable

Fixed effect	Estimate	s.e.	95% CI	χ^2^	d.f.	*P*-value
Intercept	17.68	0.32	17, 18			
Treatment				11.73	1	0.001*
Control	1.26	0.28	0.71, 1.8			
ALAN	–	–	–			
Sex				0.07	1	0.797
Female	–	–	–			
Male	0.04	0.22	−0.40, 0.48			
Brood size scaled	0.00	0.18	−0.35, 0.36	0.01	1	0.928
Date of sampling	0.31	0.18	−0.05, 0.67	2.53	1	0.111

The output shows the model estimate, standard error (s.e.), 95% confidence interval (CI), degrees of freedom (d.f.) and *P*-values. The fixed effects were Intercept, Treatment, Sex, Brood size and Date of sampling; factor levels were Control, ALAN, Female and Male. Significant *P*-values are indicated by an asterisk.

### The effects of ALAN on fCORT levels

There was no significant impact of ALAN on fCORT concentrations in either sex (Table 2, Fig. 2B).

**Table 2.** **The output** of the model with the values from the LRT, where feather **corticosterone (fCORT)** level was used as **the** response **variable**

Fixed effect	Estimate	s.e.	95% CI	χ^2^	d.f.	*P*-value
Intercept	−0.92	9.42	−19, 18			
Treatment				0.68	1	0.411
Control	–	–	–			
ALAN	−1.04	1.42	−3.8, 1.7			
SMI	0.32	0.49	−0.63, 1.3	0.44	1	0.507
Sex				0.49	1	0.485
Female	–	–	–			
Male	−1.11	1.20	−3.5, 1.2			
Brood size scaled	−1.43	0.70	−2.8, −0.07	3.45	1	0.063

The output shows the model estimate, standard error (s.e.), 95% confidence interval (CI), degrees of freedom (d.f.) and *P*-values. The fixed effects were Intercept, Treatment, SMI, Sex and Brood size; factor levels were Control, ALAN, Female and Male. Significant *P*-values are indicated by an asterisk.

### The effects of ALAN on OXY levels

The interaction between treatment and sex was not significant (χ^2^=3.086, *P*=0.079). Treatment was shown to have a significant impact on OXY levels (Table 3); birds exposed to ALAN experienced higher levels of OXY than the control birds (Fig. 2C).

**Table 3.** The output of the lmer model with the values from the LRT, where antioxidant capacity of plasma (OXY) level was used as the response variable

Fixed effect	Estimate	s.e.	95% CI	χ^2^	d.f.	*P*-value
Intercept	222.33	93.19	40, 405			
Treatment				5.66	1	0.017*
Control	–	–	–			
ALAN	28.03	11.91	4.7, 51			
SMI	−0.47	4.93	−10, 9.2	0.01	1	0.917
Sex				2.82	1	0.093
Female	–	–	–			
Male	−17.79	10.78	−39, 3.3			
Brood size scaled	4.13	6.39	−8.4, 17	0.45	1	0.504
Date of sampling	−9.11	6.89	−23, 4.4	1.83	1	0.176

The output shows the model estimate, standard error (s.e.), 95% confidence interval (CI), degrees of freedom (d.f.) and *P*-values. The fixed effects were Intercept, Treatment, SMI, Sex, Brood size scaled and Date of sampling; factor levels were Control, ALAN, Female and Male. Significant *P*-values are indicated by an asterisk.

The analysis showed a trend towards an interaction between SMI and treatment on OXY; however, this was not significant (χ^2^=3.331, *P*=0.068). For the birds exposed to ALAN, as SMI increased, OXY decreased and the opposite trend can be seen in the control group (Fig. S1).

### The effects of ALAN on MDA levels

When investigating the effects of ALAN on MDA levels, the results show that the interaction between sex and treatment was significant (Table 4). Whereas males and females exposed to ALAN had similar levels of MDA, within the control group, males had the lowest levels of MDA when compared with all other groups (Fig. 2D). The *post hoc* analysis showed that control females and control males had the biggest difference from each other in their MDA levels (mean estimate [95% confidence interval, CI]=1.629 [−0.088, 3.35]; Table S2). However, this was not statistically significant.

**Table 4.** The output of the lmer model with the values from the LRT, where malondialdehyde (MDA) level was used as the response variable

Fixed effect	Estimate	s.e.	95% CI	χ^2^	d.f.	*P*-value
Intercept	8.43	3.89	0.80, 16			
Treatment						
Control	–	–	–			
ALAN	−1.10	0.73	−2.5, 0.33			
SMI	−0.05	0.21	−0.45, 0.35	0.07	1	0.789
Sex						
Female	–	–	–			
Male	−1.63	0.63	−2.9, −0.40			
Brood size scaled	0.18	0.33	−0.47, 0.83	0.36	1	0.548
Date of sampling	−0.48	0.35	−1.2, 0.20	2.18	1	0.140
Treatment×Sex				4.27	1	0.039*
* *ALAN×Mass	1.82	0.87	0.11, 3.5			

The output shows the model estimate, standard error (s.e.), 95% confidence interval (CI), degrees of freedom (d.f.) and *P*-values. The fixed effects were Intercept, Treatment, SMI, Sex, Brood size, Date of sampling and Treatment×Sex; factor levels were Control, ALAN, Female, Male and ALAN×Mass. Significant *P*-values are indicated by an asterisk.

There was no significant effect of OXY on MDA levels (Fig. S2) and no interaction effect between OXY and treatment on MDA levels (χ^2^=1.599, *P*=0.206).

### The impacts of the physiological biomarkers on SMI

The interaction of fCORT, OXY and MDA with treatment had no statistically significant effect on SMI and so these were dropped from the model.

The results of the final model indicated that there was no significant relationship between the biomarkers measured (Table 5) and SMI. Pearson's correlation analysis revealed very weak correlations between all four biomarkers as shown in Table S3 and Fig. S3.

**Table 5.** The output of the lmer model with the values from the LRT, where SMI was used as the response variable

Fixed effect	Estimate	s.e.	95% CI	χ^2^	d.f.	*P*-value
Model 1: MDA and OXY						
Intercept	19	0.554	17, 20			
Date of sampling	0.41	0.177	0.06, 0.75	4.91	1	0.027*
MDA	−0.02	0.047	−0.11, 0.07	0.16	1	0.694
OXY	0.001	0.002	−0.002, 0.005	0.96	1	0.693
Treatment				12.36	1	<0.001*
Control	–	–	–			
ALAN	−1.2	0.257	−1.7, −0.66			
Brood size	−0.11	0.171	−0.44, 0.23	0.52	1	0.473
Sex				0.09	1	0.762
Female	–	–	–			
Male	0.05	0.213	−0.36, 0.47			
Model 2: CORT						
Intercept	18	0.792	17, 20			
Date of sampling	0.29	0.228	−0.16, 0.74	1.64	1	0.200
CORT	0.04	0.062	−0.08, 0.16	0.47	1	0.198
MDA	0.04	0.062	−0.08, 0.16	0.47	1	0.753
OXY	0.0006	0.002	−0.004, 0.005	0.06	1	0.809
Treatment				11.20	1	0.001*
Control	–	–	–			
ALAN	−1.4	0.328	−2.0, −0.73			
Sex				0.10	1	0.753
Female	–	–	–			
Male	0.08	0.276	−0.46, 0.462			

The output shows the model estimate, standard error (s.e.), 95% confidence interval (CI), degrees of freedom (d.f.) and *P*-values. The fixed effects were Intercept, Date of sampling, CORT, MDA, OXY, Treatment, Brood size and Sex; factor levels were Control, ALAN, Female and Male. The table is split into Model 1, where MDA and OXY were included as explanatory variables, and Model 2, where CORT was included as an explanatory variable; the remainder of the explanatory variables remained the same for each model. Significant *P*-values are indicated by an asterisk.

## DISCUSSION

It is well documented that organisms that are exposed to ALAN face both behavioural and physiological changes that can be detrimental to health (Bedrosian et al., 2011; Cruz et al., 2003; Jones et al., 2015; Russart and Nelson, 2018; Ziegler et al., 2021). The results of this study show that exposure to ALAN leads to reduced body condition in great tit nestlings, which may have negative consequences for fitness and survival later in life. However, there was no conclusive evidence that the changes in body condition were associated with effects of ALAN on oxidative stress or feather corticosterone levels.

### The impacts of ALAN on body condition

The findings of this study indicated that the nestlings in the ALAN treatment group had a significantly lower SMI than control birds. This result agrees with findings in previous studies where it has been shown that ALAN can have a negative impact on body condition and development in young birds (Ferraro et al., 2020; Raap et al., 2016b) and other species including cane toads (*Rhinella marina*) (Secondi et al., 2021). There may be many mechanisms behind this relationship. Nestlings have been shown to beg for food more frequently throughout the night when exposed to ALAN than when not exposed (Raap et al., 2016a); it is unlikely that this heightened begging behaviour led to an increased feeding rate by the parents as the light treatment was only used inside the nest box and the dark night-time conditions outside would not provide optimal conditions for the parents to forage. This increase in begging behaviour can be costly as it may divert important resources from physiological processes including growth and metabolism. One study was able to show that as begging behaviour intensified in captive house sparrow (*Passer domesticus*) nestlings, metabolic expenditure increased and body condition declined (Soler et al., 2014).

The decrease in SMI could also be an indirect effect of ALAN through changes in the provisioning behaviour of the parents. It has been shown that when nestboxes were exposed to ALAN it disrupted the provisioning behaviour of female tree swallows (*Tachycineta bicolor*) and resulted in nestlings being fed less frequently than control birds (Injaian et al., 2021). Furthermore, female great tits exposed to ALAN within their nest box showed delayed sleep onset and their sleep duration was reduced by half (Raap et al., 2016a). Disruption to the sleep pattern of parents while they are rearing chicks could impact parental care and as a result may negatively impact the condition of the nestlings. The impacts of a decrease in body condition prior to fledging are potentially lifelong and it has been shown that coal tits (*Periparus ater*) and great tits that leave the nest in poor condition have a lower chance of survival after fledging (Naef-Daenzer et al., 2001).

### The impact of ALAN on fCORT levels

There was no statistically significant effect of ALAN exposure on fCORT levels in great tit nestlings. The lack of an effect of ALAN was unexpected and contrasts with several studies that show exposure to ALAN can lead to increased levels of CORT including in developing birds (Alaasam et al., 2018; Grunst et al., 2020; Ouyang et al., 2015). However, only one of these studies measured CORT levels in the feathers (Grunst et al., 2020), while the remaining studies measured CORT in the plasma (Alaasam et al., 2018; Ouyang et al., 2015). CORT levels measured in plasma represent a short-term snapshot of CORT in time and may not be directly comparable with the chronic measure of CORT used in the current study.

One reason why we may be seeing no significant effect of ALAN on fCORT levels is that it may be an adaptive response of neonates where they will stop chronically high levels of CORT during early development to avoid negative consequences later in life (Romero, 2004). This has been shown in neonate mammals, where they have a reduced response to stressors in the first few days of life to protect them from the harmful effects of continuous glucocorticoid elevation (Romero, 2004). The environment where the nestlings are reared may also impact how ALAN affects CORT levels; for example, an earlier study (Dominoni et al., 2021) showed that ALAN has different effects on CORT concentration in urban and non-urban blue tit nestlings. In that study, urban birds had increased fCORT levels in response to ALAN whereas non-urban birds had decreased levels (Dominoni et al., 2021). It could be the case that birds in the urban field site are exposed to other external stressors as well as the ALAN manipulation, such as poor diet, and the combined effect of these stressors may explain the increase in feather CORT. This corresponds with the mild effect seen in the current study as the current ALAN experiment took place in non-urban environments. Another study found that developing great tits exposed to ALAN had elevated feather CORT levels, which contradicts our results. However, in that study, nestlings were not exposed to ALAN until they were 8 days old (Grunst et al., 2020). It is possible that the birds in this previous experiment were able to mount a more robust stress response when exposed to ALAN, whereas the nestlings in our study may have become habituated, having been exposed from a much earlier developmental stage. The interpretation of CORT levels must also be carefully considered. Researchers cannot say for certain whether elevated levels of CORT reflect chronic stress as CORT has many other functions including energy mobilisation (Romero, 2004). The glucocorticoid response to chronic stress may also depend on many factors including species, sex, age, season and the type of external stressor they are exposed to (Cyr and Michael Romero, 2007).

### The impacts of ALAN on oxidative stress

In this study, we found a significant effect of treatment group on OXY levels, with birds exposed to ALAN exhibiting higher OXY levels than the control groups. This suggests that ALAN exposure may cause the birds to upregulate their antioxidant defences, possibly in response to higher oxidative damage caused by ALAN. This aligns with findings from previous studies, which have shown that several bird species increase antioxidant parameters in response to environmental stressors (Casagrande and Hau, 2018; Cohen et al., 2008). However, we cannot conclude from this single biomarker alone whether this increase has a negative impact on the organism. While antioxidant defences can be energetically costly, they are also vital for mitigating oxidative stress and can serve a variety of other biological functions (Costantini et al., 2010). Diet can also play an important role in antioxidant levels, as parents may provide nestlings with antioxidant-rich food, potentially influencing the observed levels (Moller et al., 2010).

When assessing the relationship between ALAN and MDA levels, MDA levels in ALAN-exposed males and females appeared to be lower than those in control females and only slightly higher than in control males. This coupled with the higher oxidative defence found in the ALAN-exposed birds could indicate that ALAN caused higher levels of oxidative damage, which was successfully combatted by a robust antioxidant response. It is not possible to conclude that this is the case as we only collected one sample from each individual and the balance between pro-oxidants and antioxidants is a dynamic state which is constantly changing. The females in the control group had higher levels of OXY and MDA than the control males, which may suggest that females have naturally higher levels of oxidative stress than males. If this is the case, then it highlights the importance of considering sex when investigating these types of relationships.

There is evidence of sex differences in oxidative stress responses in the literature. Our study agrees with a meta-analysis that looked at oxidative stress levels across vertebrates and found that levels were usually higher in females than in males across a range of tissues (Costantini, 2018). The findings in the literature regarding whether ALAN impacts oxidative stress levels are contrasting. Several studies looking into a variety of species suggest that ALAN does not impact oxidative stress (Czarnecka et al., 2022; Dimovski and Robert, 2018; Raap et al., 2016b). One study on great tit nestlings found no effect of ALAN on a wide range of oxidative stress biomarkers including antioxidant enzymes and measures of oxidative damage (Raap et al., 2016b). That study also reported that there were no effects of sex influencing the way in which ALAN impacted oxidative stress. Another study showed that there was no effect of ALAN on lipid peroxidation or antioxidant capacity in female tammar wallabies (*Macropus eugenii*) (Dimovski and Robert, 2018). These results suggest that impacts on oxidative stress may be dependent on a range of factors including sex-specific differences and species.

It is important to highlight that one should ideally measure several biomarkers of both oxidative damage and antioxidant defence in multiple tissues (Costantini, 2019). We also need to be careful when interpreting static measures of oxidative stress as it is a dynamic process which is constantly changing, limiting our ability to capture the process at a single point in time (Azzi, 2022). It has also been discussed in several reviews that an increase of oxidative stress with age can be a major source of damage to cellular function and structure (Costantini, 2011; Yu and Chung, 2006). Organisms have therefore evolved many sophisticated mechanisms to prevent the accumulation of oxidative damage (Costantini, 2018; Miura and Endo, 2010). Therefore, it could be the case that we found limited evidence of oxidative stress in the current study because maintaining oxidative processes is critical to survival and longevity. If this is the case, then it could highlight the need for future studies to include fitness or first year survival in the experiment.

### The relationship between physiological biomarkers and body condition

No relationships were found between any of the physiological biomarkers measured and SMI. High levels of glucocorticoids have been suggested to be associated with low relative fitness as discussed in a review by Bonier (2012). Some studies have found that birds in better body condition tend to have lower levels of blood CORT (Strange et al., 2016; Vágási et al., 2020). However, other studies focusing on fCORT levels have found no correlation between fCORT and body condition (Beaugeard et al., 2019; Gormally et al., 2020). It may therefore be the case that only certain indices of CORT are correlated to body condition; for example, CORT levels measured in plasma represent a snapshot measure of CORT while CORT levels measured in feather samples represent long-term chronic levels of CORT.

High levels of oxidative stress have also been associated with negative impacts on overall fitness and survival in previous studies. One study found that Alpine swift (*Tachymartptis melba*) females laid clutches that were smaller and less likely to hatch than females that had a higher resistance to oxidative stress (Bize et al., 2008). There are limited studies that have investigated the relationship between oxidative stress and body condition, and the studies that have investigated this relationship reported contrasting results. It was shown in zebra finch nestlings that if they experienced high levels of oxidative stress in early life then this could result in phenotypic changes including a shorter tarsus length than the control birds (Romero-Haro and Alonso-Alvarez, 2020). Another study looking at captive adult parrots was able to show an association with higher levels of oxidative damage and increased body mass (Larcombe et al., 2015). They did, however, report that not all oxidative stress biomarkers were associated with body mass. It may therefore be the case that the relationship between condition and oxidative stress is a complex one that depends on various factors including life stage and environment (Boonekamp et al., 2014).

### Limitations and directions for future research

There are some limitations to the current study that must be considered and that offer scope for future research. Firstly, this study did not delve deeper into the relationship between ALAN and body condition. It would be beneficial for future studies to investigate the mechanisms behind this relationship; for instance, whether it was a consequence of a direct effect due to the increased energy expenditure of the begging nestlings or an indirect effect due to changes in the provisioning behaviour of the parents. This type of behaviour could be measurable using cameras set up within the nest box (Grzędzicka, 2017; McCowan and Griffith, 2014; Mutzel et al., 2019; Raap et al., 2016a; Soler et al., 1999).

Secondly, our study was limited to a single field season. While incorporating multiple years of data would have strengthened our results by accounting for external factors such as weather variability between years, multi-year studies are not always feasible. Many previous field studies have also relied on single-season data (Dominoni et al., 2021; Grunst et al., 2019, 2020; Raap et al., 2016c, 2017a). Combined, these studies contribute to a growing body of research that can help identify broader trends and mechanisms. These individual studies may support future comprehensive meta-analysis on this topic. However, in order to assess annual variation, multiple field seasons would be necessary in future studies.

Lastly, our study was limited by a relatively small sample size. As some interactions showed weak or marginal significance, these results should be interpreted with caution. Further studies with larger sample sizes are therefore needed to confirm these patterns, which would strengthen the findings in the current study.

### Conclusions

As urbanisation continues to increase globally, wildlife is being exposed to increasing amounts of urban stressors including ALAN. Therefore, it is becoming more important to understand the impacts of these urban stressors on the health and fitness of the wildlife that is exposed to them. The results of this study show that the body condition of great tit nestlings is negatively impacted by exposure to ALAN, which could have negative consequences for their future fitness and survival. However, this study provided no evidence of ALAN having effects on the fCORT levels of the nestlings. We did observe a sex-dependent effect of ALAN on oxidative stress, although it remains unclear whether this change reflects a negative impact. These findings highlight the complex, variable nature of the impact of ALAN on physiological effects and reinforce the need for future studies to include more biomarkers of health to better understand what aspects of health and physiology are impacted by ALAN. It is also important for future research to focus on the mechanisms behind the impact of ALAN on body condition. If we can better understand this relationship, then it would help future conservation efforts in urban areas to be better targeted. There is also a need for future studies to further investigate the implications of ALAN for oxidative balance, taking into consideration differences between sexes.

## Supplementary Material

10.1242/jexbio.249926_sup1 Supplementary information
