# Supplementary material for: Artificial light at night weakens body condition but does not negatively affect physiological markers of health in great tits
Source: J Exp Biol. 2025 Jul 7;228(13):jeb249926. doi: 10.1242/jeb.249926 (PMC12276810; doi:10.1242/jeb.249926)
Supplement: Supplementary information [file jexbio-228-249926-s1.pdf]

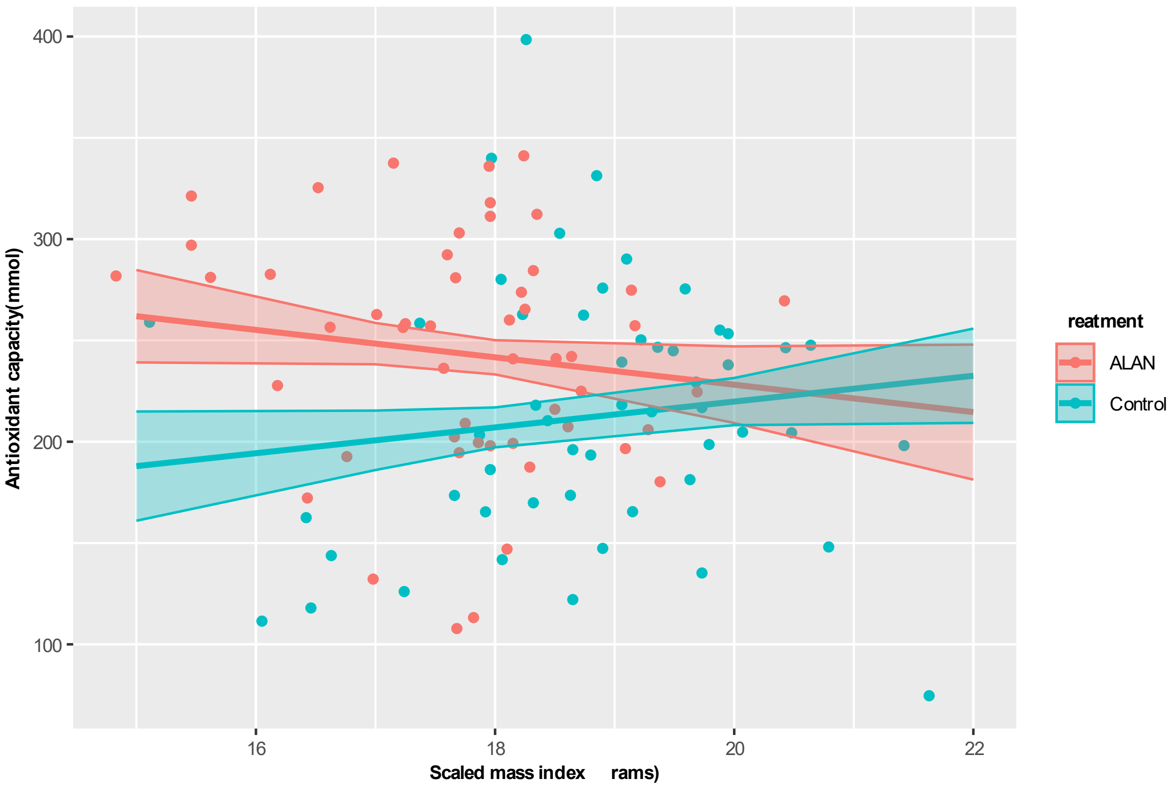

Fig. S1. OXY affected by SMI and treatment group. This effect plot shows the relationship 37 between Antioxidant capacity of plasma (OXY) and scaled mass index (SMI) in the different 38 treatment groups. The raw data points (blue represent control group and red represents the 39 ALAN group) are shown along with solid regression lines (blue represents the control group and 40 red represents the ALAN group) which was calculated using the predicted values from the LMM 41 model. The shaded ribbon around regression line shows the 95% confidence intervals.

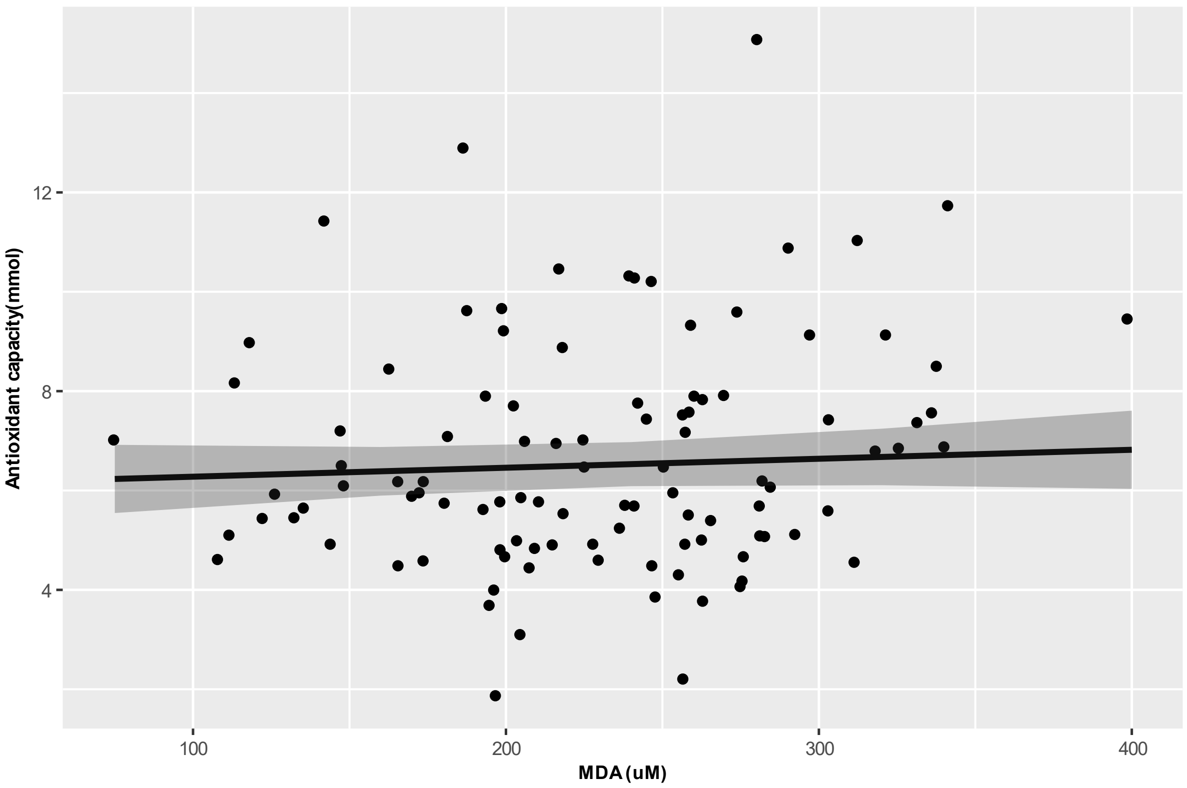

Fig. S2. MDA levels not dependent on OXY levels. The scatterplot shows the relationship 60 between MDA levels and OXY across all individuals. The raw data points are shown along with a 61 solid regression line which was calculated using the predicted values from the lmer model. The shadow around the regression line shows the 95% confidence intervals calculated for the predicted values.

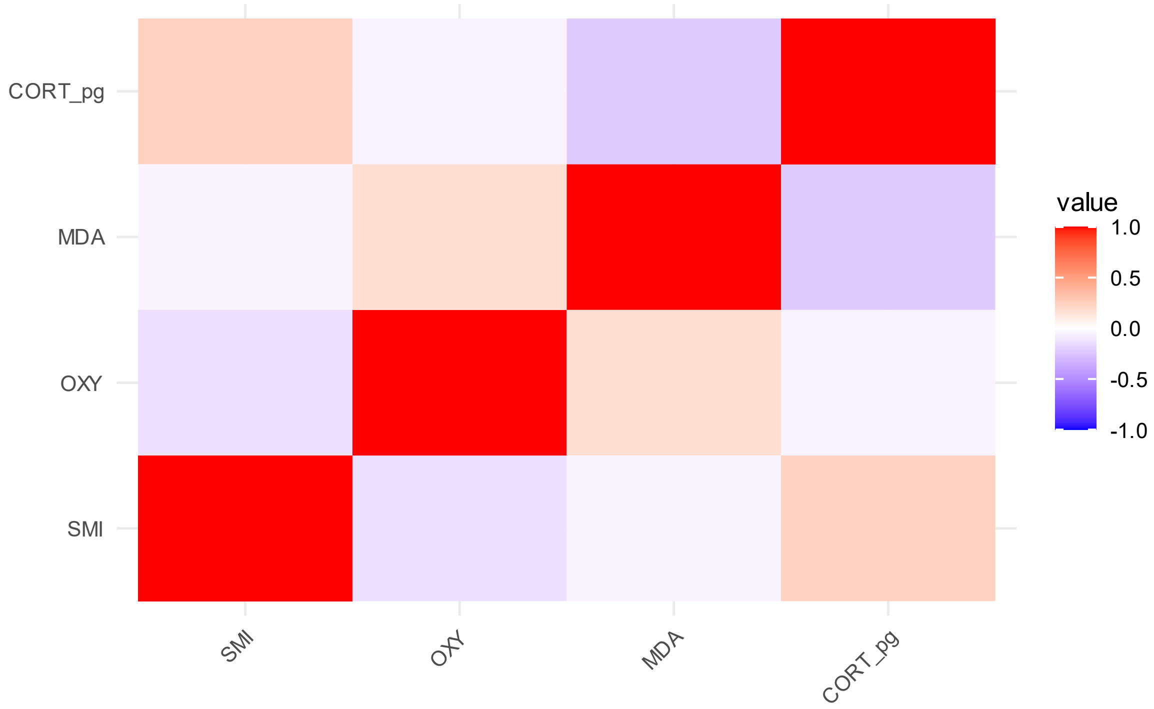

Fig. S3. This heatmap visually presents the Pearson's correlation values between biomarkers. The intensity of the colour represents the strength of the correlation, with red indicating a strong positive correlation and blue showing a strong negative correlation.

Table S1. Number of control and experimental boxes at each study location along with the coordinates of each location.

Site name	Location		No of experimental boxes	No of control boxes
	Latitude	Longitude		
Cashel	56.1093	-4.5779	3	2
Drymen Forest	56.0728	-4.4526	1	2
Killearn Forest	56.0410	-4.3881	2	1
Strathblane Forest	55.9887	-43881	1	1
Mugdock Park	55.9624	-4.3179	1	2
Sallochy	56.1238	-4.6008	3	4
SCENE	56.1299	-4.6171	3	3

Journal of Experimental Biology • Supplementary information

Table S2. The results of the post hoc analysis showing the pairwise comparisons of treatment and sex and their impact on MDA levels. The P values are tukey adjusted.

	Est	SE	P	Ci low	Ci Upp
**Control F – ALAN F**	1.101	0.755	0.471	-0.910	3.11
**ALAN F – ALAN M**	-0.193	0.610	0.989	-1.791	1.40
**ALAN F – Control M**	0.528	0.803	0.912	-1.600	2.66
**Control F – ALAN M**	0.907	0.734	0.608	-1.052	2.87
**Control F – Control M**	1.629	0.656	-0.069	-0.088	3.35
**Control M – ALAN M**	-0.721	0.783	0.793	-2.799	1.36

Table S3. This table shows the Pearson correlation coefficients between the biomarkers measured in the study. Values range from -1 (perfect negative correlation) to +1 (perfect positive correlation)

	SMI	OXY	MDA	fCORT
SMI	1	-0.125	-0.047	0.241
OXY	-0.125	1	0.17	-0.051
MDA	-0.047	0.17	1	-0.222
fCORT	0.241	-0.051	-0.222	1

Journal of Experimental Biology • Supplementary information
